# Taurolithocholic acid promotes intrahepatic cholangiocarcinoma cell growth via muscarinic acetylcholine receptor and EGFR/ERK1/2 signaling pathway

**DOI:** 10.3892/ijo.2015.2939

**Published:** 2015-03-27

**Authors:** SUMET AMONYINGCHAROEN, TAWIT SURIYO, APINYA THIANTANAWAT, PIYAJIT WATCHARASIT, JUTAMAAD SATAYAVIVAD

**Affiliations:** 1 Chulabhorn Graduate Institute, Bangkok 10210, Thailand; 2 Laboratory of Pharmacology, Chulabhorn Research Institute, Bangkok 10210, Thailand; 3 Center of Excellence on Environmental Health and Toxicology, Office of Higher Education Commission, Ministry of Education, Bangkok 10400, Thailand

**Keywords:** intrahepatic cholangiocarcinoma, bile acids, taurolithocholic acid, mAChR, EGFR

## Abstract

Cholangiocarcinoma (CCA) is a malignant cancer of the biliary tract and its occurrence is associated with chronic cholestasis which causes an elevation of bile acids in the liver and bile duct. The present study aimed to investigate the role and mechanistic effect of bile acids on the CCA cell growth. Intrahepatic CCA cell lines, RMCCA-1 and HuCCA-1, were treated with bile acids and their metabolites to determine the growth promoting effect. Cell viability, cell cycle analysis, EdU incorporation assays were conducted. Intracellular signaling proteins were detected by western immunoblotting. Among eleven forms of bile acids and their metabolites, only taurolithocholic acid (TLCA) concentration dependently (1–40 μM) increased the cell viability of RMCCA-1, but not HuCCA-1 cells. The cell cycle analysis showed induction of cells in the S phase and the EdU incorporation assay revealed induction of DNA synthesis in the TLCA-treated RMCCA-1 cells. Moreover, TLCA increased the phosphorylation of EGFR, ERK 1/2 and also increased the expression of cyclin D1 in RMCCA-1 cells. Furthermore, TLCA-induced RMCCA-1 cell growth could be inhibited by atropine, a non-selective muscarinic acetylcholine receptor (mAChR) antagonist, AG 1478, a specific EGFR inhibitor, or U 0126, a specific MEK 1/2 inhibitor. These results suggest that TLCA induces CCA cell growth via mAChR and EGFR/EKR1/2 signaling pathway. Moreover, the functional presence of cholinergic system plays a certain role in TLCA-induced CCA cell growth.

## Introduction

Cholangiocarcinoma (CCA) is a malignant tumor arising from the biliary tract epithelium, cholangiocyte. The conditions associated with chronic biliary tract inflammation such as primary sclerosing cholangitis (PSC), parasitic infection, viral infection and chemical carcinogen exposure, are major risk factors associated with the development of CCA (1). However, the specific etiology and molecular pathogenesis of CCA remain to be comprehensively elucidated.

Bile acids are endogenous substances which play a role in several important physiological processes (2). Bile acid exposure has been reported to be associated with an increasing incidence of gastrointestinal cancers (3). Bile acids inducing cancer cell proliferation via epidermal growth factor receptors (EGFR), Farnesoid X receptors (FXR), sphingosine 1-phosphate receptor 2 (S1PR2), and G-protein-coupled bile acid receptor 1 (TGR5) have been associated with many types of cancer such as colon, liver and uterus (4–7). Furthermore, deoxycholic acid (DCA), lithocholic acid (LCA) and their taurine conjugates stimulate colon cancer cell proliferation through muscarinic acetylcholine receptor subtype M3 (M3 mAChR) (8–10). However, our knowledge of the roles of bile acids on CCA cell growth is limited and more study is needed.

Cholinergic systems are functionally present on certain types of cancer cells including lung, colon, cervix, prostate and breast cancers (11–15). The cholinergic system plays a role in the regulation of important cell functions, including proliferation, migration, cell-to-cell communication and other features critical for cancer progression (16,17). More importantly, it has been shown that the expression of M3 mAChR plays a key role in the proliferation and metastasis of CCA (18). Furthermore, the cholinergic denervation of the liver results in the induction of cell death and impairs proliferative response of cholangiocyte to cholestasis (19). In the present study, we focused on the effects of different bile acids and their metabolites on the growth of two different intrahepatic CCA cell lines. HuCCA-1 cells were obtained from a Thai-CCA patient with a history of parasitic infection (*Opisthorchis viverrini*), while RMCCA-1 cells were established from a Thai-CCA patient with a history of non-parasitic infection. The mechanistic effect of bile acids in CCA growth was also investigated.

## Materials and methods

### Materials

Eleven forms of bile acids and their metabolites were purchased from Sigma-Aldrich (St. Louis, MO, USA). These included cholic acid (CA, purity ≥98%), chenodeoxycholic acid (CDCA, purity ≥97%), deoxycholic acid (DCA, purity ≥98%), lithocholic acid (LCA, purity ≥97%), glycocholic acid (GCA, purity ≥97%), glycochenodeoxycholic acid (GCDCA, purity ≥97%), glycodeoxycholic acid (GDCA, purity ≥97%), taurocholic acid (TCA, purity ≥95%), taurochenodeoxycholic acid (TCDCA, purity ≥95%), taurodeoxycholic acid (TDCA, purity ≥97%), and taurolithocholic acid (TLCA, purity ≥97%). Carbachol and oxotremorine-M were also purchased (Sigma-Aldrich). AG 1478 was obtained from Calbiochem (Germany). U 0126 was ordered from Cell Signaling Technology (Beverly, MA, USA).

### Cell culture

The human intrahepatic CCA cell lines, including HuCCA-1 and RMCCA-1 derived from bile duct tumor mass of Thai CCA patients, were established and kindly provided by Professor Stitaya Sirisinha (20), and Dr Kawin Leelawat (21), respectively. Both HuCCA-1 and RMCCA-1 cells were grown in Ham’s F-12 medium (Gibco, Carlsbad, CA, USA), supplemented with 10% FBS (JR Sientific, Inc., Woodland, CA, USA), 2 mM L-glutamine, 100 U/ml penicillin and 100 μg/ml streptomycin (Gibco), at 37°C in a 5% CO_2_ humidified atmosphere. Human neuroblastoma SH-SY5Y cells obtained from American Type Culture Collection (ATCC) were grown in a 1:1 mixture of minimum essential medium (MEM) (Gibco) and Ham’s F12 medium supplemented with 10% FBS, 2 mM L-glutamine, 100 U/ml penicillin and 100 μg/ml streptomycin, and cultured in 5% CO_2_ at 37°C humidified atmosphere.

### MTT assay

Cell viability was measured by a quantitative colorimetric assay (MTT) (1-(4,5-Dimethylthiazol-2-yl)-3,5-diphenylformazan) (Sigma-Aldrich) showing the mitochondrial activity of living cells. Briefly, human CCA cells were plated in 96-well plates (1×10^4^ cells/well) and cultured overnight for attachment. The next day, cell synchronization was performed by incubating in serum-free medium for 24 h. The synchronized cells were treated with different bile acids and their metabolites for 48 h in serum-free medium, in order to reduce growth promoting effects of the growth factor and steroid hormones present in the serum. Thereafter, the medium was aspirated, and 100 μl of 500 μg/ml of MTT in serum-free medium was added to each well. Cells were incubated with MTT for 4 h; next, cells were lysed by dimethyl sulfoxide. When the formazan crystals were completely dissolved, the optical density (OD) was measured at 570 nm and reference wavelength at 650 nm, using a SpectroMax M3 microplate reader (Molecular Devices, Sunnyvale, CA, USA).

### PrestoBlue cell viability assay

PrestoBlue reagent is quickly reduced by metabolically active cells, providing a quantitative measure of viability and cytotoxicity. CCA cells were processed as in the previous MTT assay. At the end of the respective incubation period 24–48 h, cell viability was determined by adding 10 μl of 10x PrestoBlue Cell Viability reagent (Invitrogen, Carlsbad, CA, USA) and incubating at 37°C for 30 min. The fluorescence was determined at 560 nm excitation/590 nm emission using SpectroMax M3 microplate reader, and expressed as the percentage of cell viability of the control.

### EdU incorporation assay

The cell proliferation was determined by the incorporation of 5-ethynil-2-deoxyuridine (EdU) into newly synthesized DNA stand, using a Click-iT EdU microplate assay kit (Invitrogen) according to the manufacturer’s instructions. Briefly, cells were processed as previously described in the cell viability assay. After 24-h treatment with bile acid, 10 μl of 10x EdU working solution was added to each well to make the final concentration of 10 μM. The incorporation time was 4 h. Then the incorporated EdU in DNA was coupled with Oregon Green-azide dye, and subsequently incubated with horseradish peroxidase-labeled anti-Oregon Green antibody and Amplex UltraRed. The fluorescence was determined at 490 nm excitation/585 nm emission using SpectroMax M3 microplate reader, and expressed as the percentage of cell proliferation of the control.

### Cell cycle analysis

CCA cells were plated into 6-well plates (1×10^6^ cells/well) and cultured overnight. Cells were processed as stated in the previous cell viability assay. After 24-h treatment, cells were trypsinized and washed with cold phosphate buffer saline (PBS). Subsequently, cells were fixed by using 70% ethanol at 4°C for 1 h and then washed with cold PBS. Cells were stained by adding 1 ml of propidium iodide solution containing 50 μg/ml propidium iodide (Sigma-Aldrich) and 0.5 ng/ml RNAse (Sigma-Aldrich). Analysis was performed with a BD FACSCanto™ flow cytometer (BD Biosciences, San Diego, CA, USA) and cell cycle distribution was analyzed by ModFit LT software (Verity House Software, Topsham, ME, USA).

### Western blotting

The cells were processed as in the above described cell viability assay. At the end of the respective incubation period, cells were lysed in lysis buffer containing 10 mM Tris (pH 7.4), 150 mM NaCl, 1% Triton X-100, 1 mM PMSF, 1 mM Na_3_VO_4_, 20 mM NaF and 1X protease inhibitor cocktail set I (Calbiochem). Cell lysates were sonicated and incubated at 4°C for 30 min before being centrifuged at 16,000 × g for 15 min at 4°C. The concentration of protein was determined by using Bradford reagent (Bio-Rad, Hercules, CA, USA). The protein (50 μg) was electrophoresed onto a 7.5% SDS-polyacrylamide gel, in a Mini-Protean II system (Bio-Rad). The separated protein bands were transferred onto a nitrocellulose membrane using a Bio-Rad Mini Trans-Blot cell. The nitrocellulose membrane was incubated in blocking buffer (5% non-fat dry milk in TBST buffer [10 mM Tris-HCl pH 8.0, 150 mM NaCl, and 0.05% Tween-20)] for 1 h at room temperature, followed by overnight incubation at 4°C with the primary antibody. The antibodies against cyclin D1 (1:1,000), phospho-ERK1/2 (1:2,000) and total ERK1/2 (1:2,000) were obtained from Cell Signaling Technology and antibodies against phospho-EGFR (1:1,000), EGFR (1:1,000), COX-2 (1:2,000), CHT (1:2,000), ChAT (1:2,000), AChE (1:1,000), M3 mAChR (1:1,000) and α7 nAChR (1:1,000) were purchased from Santa Cruz Biotechnology (Santa Cruz, CA, USA). The membrane was washed three times for 10 min each with TBST, and then incubated for 2 h at room temperature with appropriate secondary antibody conjugated with horseradish peroxidase. The protein bands stained with the antibodies were visualized by using enhanced chemiluminescence (ECL) (GE Healthcare, UK). The intensity of protein bands was quantified by Image Quant TL software (GE Healthcare).

### Statistical analysis

The experiments were performed in triplicate, and the results are expressed as the means ± SEM. For individual comparison, statistical analysis was performed using a two-tailed Student’s t-test. Multiple comparisons were performed using one-way analysis of variance (ANOVA) followed by the Student-Newman-Keuls test. Data with statistical values of p<0.05 are considered as statistically significant.

## Results

### The existence of inflammation marker, COX-2 and cholinergic components in HuCCA-1 and RMCCA-1 cells

**Figure 1:**
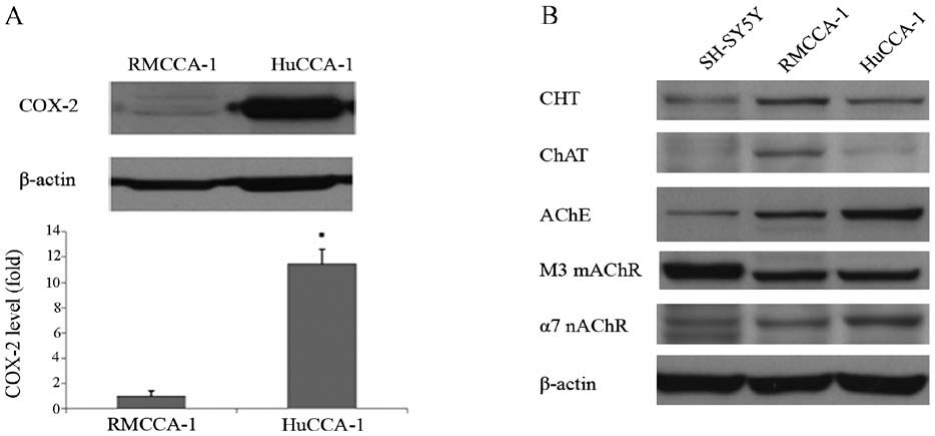
Comparison of COX-2 and cholinergic components in HuCCA-1 and RMCCA-1. (A) Western blotting of COX-2 protein and band density compared between HuCCA-1 and RMCCA-1 (^*^p<0.05 as compared with RMCCA-1). (B) Western blotting of cholinergic component proteins in SH-SY5Y (used as a positive control), RMCCA-1 and HuCCA-1 cells; CHT, choline transporter; ChAT, choline acetyltransferase; AChE, acetylcholine esterase; M3 mAChR, M3 muscarinic acetylcholine receptor; α7 nAChR, α7 nicotinic acetylcholine receptor.

The endogenous background levels of inflammation and cholinergic components in CCA cells were determined. The results showed that HuCCA-1 cells have higher expression level (11.5 times) of COX-2, a key inflammatory marker protein than RMCCA-1 cells (Fig. 1A). Furthermore, the existence of cholinergic systems was also different among these two CCA cell lines. All of the cholinergic components including choline transporter (CHT), choline acetyltransferase (ChAT), acetylcholine esterase (AChE), M3 muscarinic acetylcholine receptor (M3 mAChR) and α7 nicotinic acetylcholine receptor (α7 nAChR) were detected in both HuCCA-1 and RMCCA-1 cells (Fig. 1B). Note that, RMCCA-1 cells expressed higher levels of CHT and ChAT than HuCCA-1 cells. Respectively, AChE and α7 nAChR expression was lower in RMCCA-1 than HuCCA-1 cells. It is interesting to note that both CCA cell lines expressed higher levels of the cholinergic components than dopaminergic/cholinergic neuroblastoma SH-SY5Y cells, except the M3 mAChR.

### TLCA increases viability of RMCCA-1 cells

**Figure 2:**
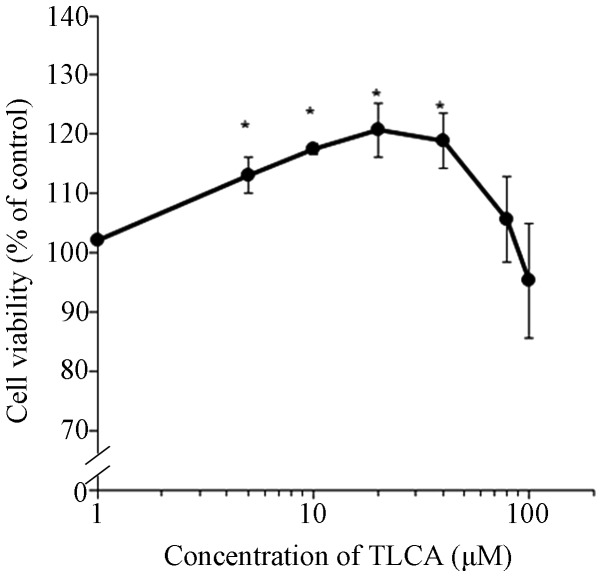
PrestoBlue cell viability assay of RMCCA-1 cells treated with TLCA at 1, 5, 10, 20, 40, 80 and 100 μM for 48 h (^*^p<0.05 as compared with control)

After treatment with bile acids and metabolites for 48 h, cell viability was determined by MTT assay. As shown in Table I, most of primary bile acids, secondary bile acids and their glycine-conjugated at the highest-tested concentration (100 μM) significantly decreased the viability of CCA cells, except CA and GCDCA in RMCCA-1 cells. Tauroline-conjugated bile acids at the highest-tested concentration did not significantly decrease the viability of CCA cells, except TCGCA and TLCA in HuCCA-1, and TLCA in RMCCA-1 cells. Primary bile acid CDCA showed higher cytotoxic effect to the CCA cells than another primary bile acid CA. In addition, secondary bile acids, including DCA and LCA, showed higher cytotoxic effects to the CCA cells than their primary bile acids. It is interesting to find that among the 11 forms of bile acid and their metabolites only low tested concentration (0.1–10 μM) of TLCA increased the viability of RMCCA-1 cells. However, at high concentration decreasing of cell viability was observed. The effect of TLCA to increase cell viability was confirmed by using the PrestoBlue cell viability assay. The results obtained from PrestoBlue cell viability assay showed a similar pattern of MTT assay with a higher sensitivity (Fig. 2). TLCA significantly increased the RMCCA-1 viable cells starting at 5 μM until 40 μM. However, concentrations >40 μM of TLCA caused decreasing trends of cell viability. Note that TLCA at the concentration of 10 μM was selected for further study.

### TLCA induces RMCCA-1 cell growth

**Figure 3:**
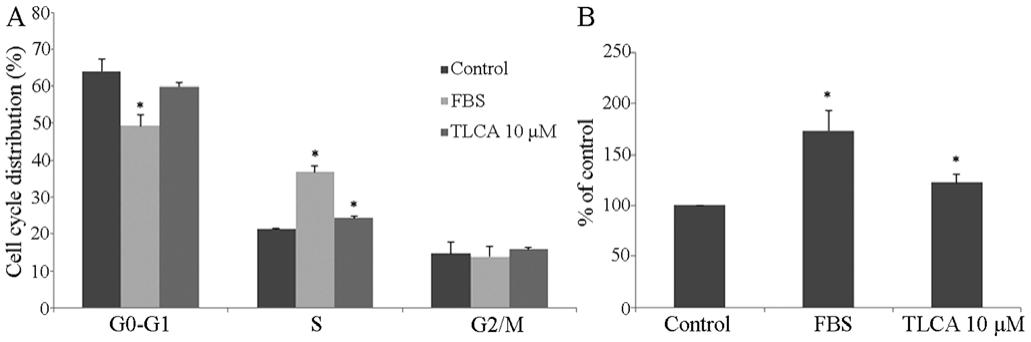
TLCA effects on the growth of RMCCA-1 cells. RMCCA-1 cells were treated with fetal bovine serum (10% v/v) or TLCA (10 μM) for 24 h and then stained with propidium iodide. Cell cycle was analyzed by ModFit LT software. (A) Cell distribution of cell cycle analysis. (B) RMCCA-1 cell proliferation was determined by using EdU incorporation assay (^*^p<0.05 as compared with control).

**Figure 4:**
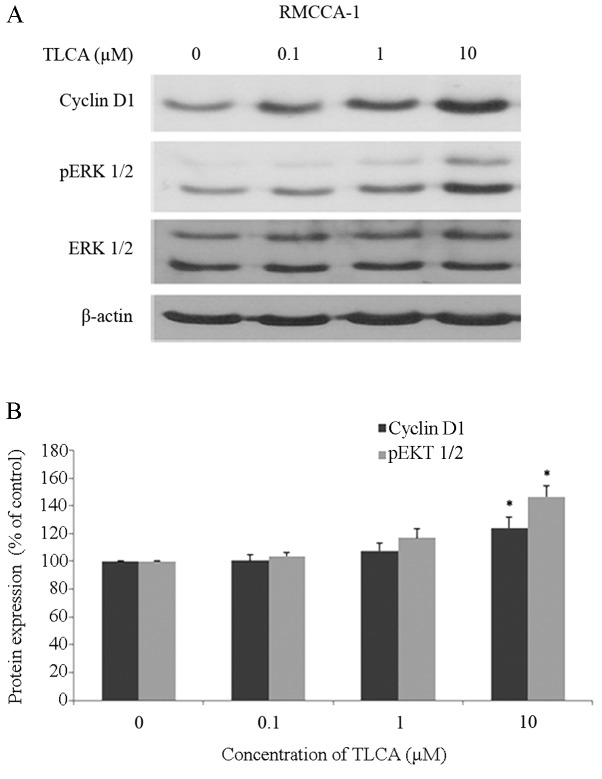
Effect of TLCA on cyclin D1 and pERK 1/2. (A) Western blot bands of cyclin D1, pERK 1/2, ERK 1/2 and β-actin protein of RMCCA-1 cells treated with TLCA for 48 h. (B) The band density ratio of cyclin D1 and pERK 1/2 protein was determined (^*^p<0.05 as compared with control).

**Figure 5:**
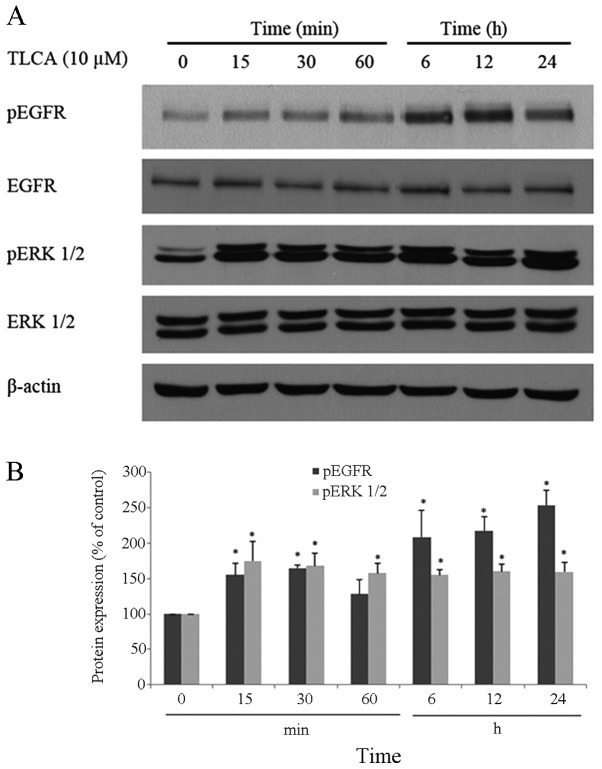
Effects of TLCA on EGFR and ERK. (A) W estern blot bands of pEGFR, EGFR, pERK 1/2, ERK 1/2 and β-actin protein of RMCCA-1 cells treated with TLCA 10 μM at varying times. (B) The band density ratio of pEGFR and pERK 1/2 protein was determined (^*^p<0.05 as compared with time 0 min).

Our results revealed that TLCA increased the RMCCA-1 cell viability in serum-free conditions, suggesting the growth promoting effect of TLCA. Therefore, further study was conducted to investigate the effect of TLCA on cell cycle and DNA synthesis of RMCCA-1 cells. The results showed that 10 μM of TLCA and the positive control (10% FBS) treatment for 24 h significantly increased the percentage of S-phase cell subpopulation (Fig. 3A). Moreover, the effect of TLCA on the proliferation of RMCCA-1 was detected by the EdU incorporation assay. TLCA at the concentration of 10 μM and 10% of FBS treatment significantly increased cell proliferation by 22.3 and 73.8%, respectively, when compared with the control (Fig. 3B). These results indicate that the rise in TLCA-treated cell viability was caused by cell proliferation. Furthermore, cyclin D1 and phosphorylated-ERK 1/2 of RMCCA-1 cells treated with TLCA were increased in a concentration-dependent pattern, a statistically significant difference at the concentration of 10 μM (Fig. 4). Additionally, time course study showed that 10 μM of TLCA continuously increases the phosphorylation of ERK 1/2 and EGFR (Fig. 5), indicating the activation of these proteins. The activation of ERK 1/2 was observed at 15 min following the TLCA treatment, and this activation remained consistent throughout the exposure time (24 h). The activation of EGFR was also found at 15 min after treatment, and this activation was time-dependent. Despite induction of their phosphorylated forms, the levels of total forms of ERK 1/2 and EGFR were not changed at any time on the TLCA treatment. These results indicate that TLCA induces cell growth and activates the phosphorylation of both EGFR and ERK 1/2 in RMCCA-1 cells.

### Muscarinic acetylcholine receptors involved in TLCA-activated RMCCA-1 cell growth

**Figure 6:**
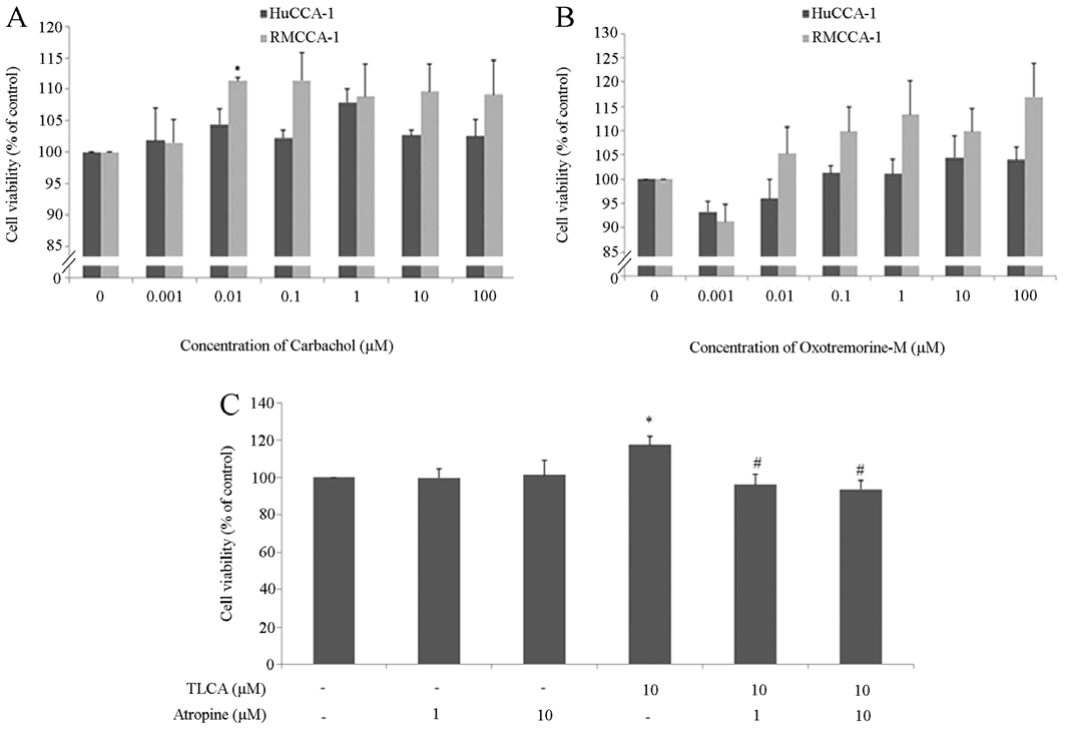
Effect of acetylcholine receptor agonists and TLCA on CCA cell lines. (A) PrestoBlue cell viability of HuCCA-1 and RMCCA-1 cells treated with carbachol for 48 h. (B) PrestoBlue cell viability of HuCCA-1 and RMCCA-1 cells treated with oxotremorine for 48 h. (C) PrestoBlue cell viability of RMCCA-1 cells treated with TLCA and/or atropine for 48 h (^*^p<0.05 as compared with control; ^#^p<0.05 as compared with TLCA 10 μM).

Cholinergic system plays an important role in cholangiocyte biology including modulating growth, apoptosis, and secretion of cholangiocytes (22). Importantly, mAChR subtype M3 (M3 mAChR) plays a key role in the proliferation and metastasis of CCA (18). To investigate the functional role of cholinergic system in CCA cell growth, HuCCA-1 and RMCCA-1 cells were treated with carbachol, which is a stable cholinergic receptor agonist or oxotremorine-M (Oxo-M), a specific mAChR agonist, in a serum-free condition; then cell viability was determined after 48 h of exposure. The results showed that 0.01 μM of carbachol significantly increased the growth of RMCCA-1 cells (Fig. 6A). Carbachol at the higher concentration (0.1–100 μM) also increased the growth of RMCCA-1 cells, however significant difference to the control group was not observed. Moreover, none of the tested concentration of carbachol (0.001–100 μM) showed a growth promoting effect in HuCCA-1 cells. Furthermore, Oxo-M (0.01–100 μM) also slightly increased the growth of RMCCA-1 cells but this effect was not found in HuCCA-1 cells (Fig. 6B). We observed that the increase in cell viability induced by two cholinergic agonists, HuCCA-1 was less responsive than RMCCA-1. The different results observed in RMCCA-1 and HuCCA-1 cell lines may be due to the difference in the basal cholinergic function of these two cell lines. However, these results may suggest that cholinergic system plays some role in RMCCA-1 cell growth.

To investigate the role of mAChR in TLCA-induced RMCCA-1 cell growth, RMCCA-1 cells were treated with 10 μM of TLCA and/or 1, 10 μM of atropine, which is a nonselective antagonist of mAChR for 48 h. The results showed that atropine by itself did not alter the growth of RMCCA-1 cells, whereas atropine completely mitigated the growth promoting effect of TLCA (Fig. 6C). This result indicates that mAChR is involved in TLCA-stimulated RMCCA-1 cell growth.

**Figure 7:**
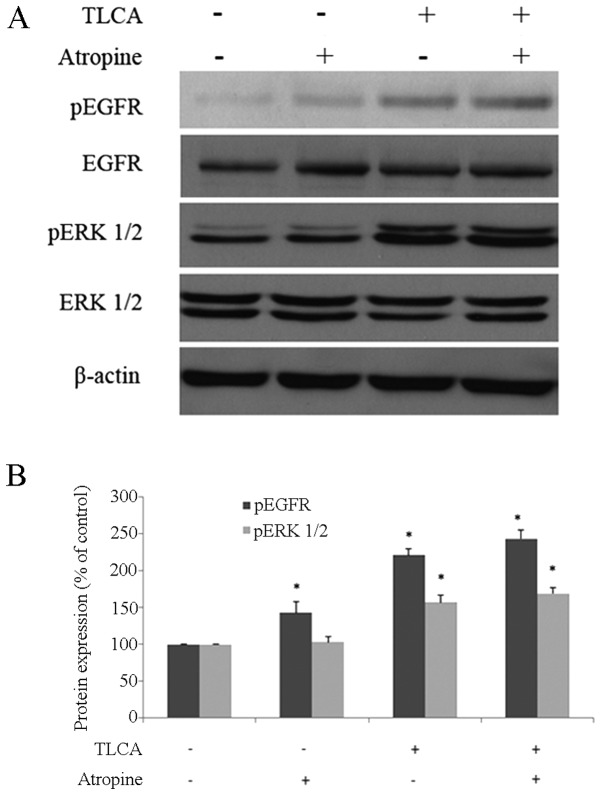
M3 AChR did not transactivate the EGFR. (A) Western blot bands of pEGFR, EGFR, pERK 1/2, ERK 1/2 and β-actin protein of RMCCA-1 cells treated with TLCA 10 μM and/or atropine 10 μM at 1 h. (B) The band density ratio of pEGFR and pERK 1/2 protein (^*^p<0.05 as compared with control).

It has been reported that some forms of bile acids, including DCA, LCA, GDCA, TDCA, GLCA and TLCA, induced growth of colon cancer cells, through the M3 mAChR-transactived EGFR signaling pathway (10). Next, we investigated the role of M3 mAChR in TLCA-induced activation of EGFR in RMCCA-1. The cells were treated with 10 μM of TLCA or 10 μM of atropine for 1 h before western blotting. For combined-treatment, RMCCA-1 cells were pre-treated with 10 μM of atropine for 30 min before being co-exposed with 10 μM of TLCA. The result showed that phosphorylated-EGFR significantly increased with a single-treatment of atropine or TLCA while combined-treatment did not reduce the activation of EGFR (Fig. 7). Furthermore, the increase of ERK 1/2-phosphorylated form by TLCA was not reduced in the atropine/TLCA co-treatment group (Fig. 7).

### TLCA induces RMCCA-1 cell growth through activation of EGFR/ERK1/2 signaling pathway

**Figure 8:**
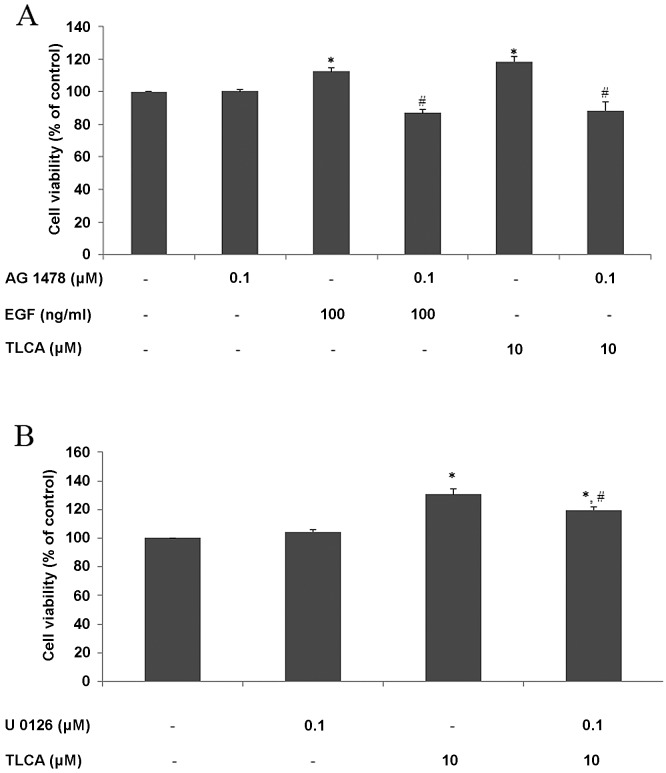
TLCA effects on EGFR and MAP kinase of RMCCA-1 cells. PrestoBlue cell viability assay was used. (A) RMCCA-1 cells were treated with EGF 100 ng/ml or TLCA 10 μM with or without co-incubation AG 1478 for 24 h. (B) RMCCA-1 cells were treated with TLCA 10 μM and/or U 0126 for 48 h (^*^p<0.05 was compared with control; ^#^p<0.05 as compared with EGF 100 ng/ml or TLCA 10 μM).

EGFR is a membrane receptor that plays an important role in regulating cell proliferation and death. The hypothesis that TLCA induces CCA cell growth through activation of EGFR was tested using AG 1478, which is a specific inhibitor of EGFR. RMCCA-1 cells were pretreated with AG 1478 for 30 min before being treated with EGF or TLCA for 24 h, and cell viability was detected by PrestoBlue reagent. The results showed that 100 ng/ml of EGF or 10 μM of TLCA increased cell viability to 113 and 118% of control, respectively. Furthermore, pre- and co-treatment with AG 1478 mitigated growth promoting effects of both EGF and TLCA. Moreover, AG 1478 by itself did not affect cell viability (Fig. 8A). These results demonstrate that the activation of EGFR is involved in TLCA-induced RMCCA-1 cell growth.

To investigate the involvement of MAP kinase pathway in TLCA-induced CCA cell growth, RMCCA-1 cells were pretreated with 0.1 μM of U 0126, which is a MEK 1/2 inhibitor, for 30 min before being treated with TLCA for 48 h. The results showed that 0.1 μM of U 0126 did not affect RMCCA-1 cell viability, but at this concentration, U 0126 significantly attenuated the effects of TLCA-induced RMCCA-1 cell viability at TLCA 10 μM (Fig. 8B). These results suggested that MAP kinase pathway is involved in TLCA-induced RMCCA-1 cell growth.

**Figure 9:**
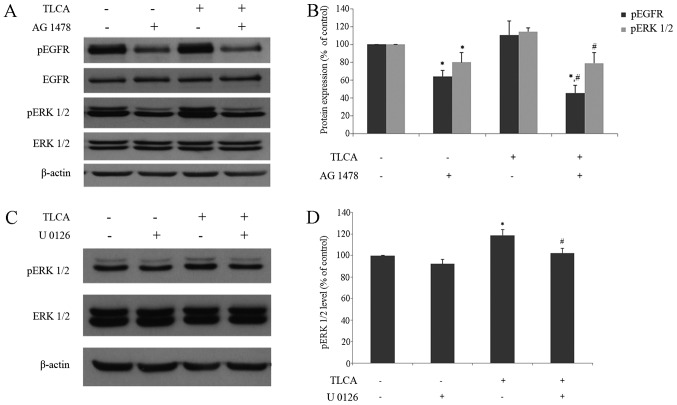
TLCA activates MAP kinase via the EGFR receptor. (A) Western blot bands of pEGFR, EGFR, pERK 1/2, ERK 1/2 and β-actin protein of RMCCA-1 cells treated with TLCA 10 μM and/or AG 1478 0.1 μM at 6 h. (B) The band density ratio of pEGFR and pERK 1/2 protein. (C) Western blot bands of pERK 1/2, ERK1/2, and β-actin protein of RMCCA-1 cells treated with TLCA 10 μM and/or U 0126 0.1 μM at 24 h. (D) The band density ratio of pERK 1/2 protein (^*^p<0.05 as compared with control; ^#^p<0.05 as compared with TLCA 10 μM).

Western blotting of TLCA and/or AG 1478 treated RMCCA-1 cells was performed in order to investigate the role of EGFR signaling pathway on TLCA-induced ERK 1/2 activation. For a single treatment, RMCCA-1 cells were treated with either 10 μM of TLCA, or 0.1 μM of AG 1478 for 6 h. For combined-treatment, RMCCA-1 cells were pre-treated with 0.1 μM of AG 1478 for 30 min before being co-exposed with 10 μM of TLCA. The result showed that phosphorylated-EGFR was increased in TLCA single treatment, whereas combined-treatment of TLCA with AG 1478 significantly reduced TLCA-induced phosphorylation of EGFR (Fig. 9B). In addition, the increase of ERK 1/2-phosphorylated form by TLCA was also significantly reduced in AG 1478/TLCA co-treatment group (Fig. 9B). It should be noted that AG 1478 treatment by itself dramatically reduced activation of both EGFR and ERK1/2. These results demonstrated that TLCA induces activation of ERK1/2 signaling pathway in part via EGFR. Furthermore, U 0126 was used to support the signaling cascade via MAP kinase pathway. RMCCA-1 cells were treated with TLCA 10 μM and/or U 0126 0.1 μM for 24 h. For combined-treatment, RMCCA-1 cells were pre-treated with 0.1 μM of U 0126 for 30 min, before being co-exposed with 10 μM of TLCA. The results show that TLCA significantly increased phosphorylated-ERK 1/2 protein, while combined-treatment of TLCA and U 0126 significantly reduced phosphorylated-ERK 1/2 protein, when compared with TLCA alone (Fig. 9D). These results suggested that TLCA induces RMCCA-1 cell growth through MAP kinase signaling pathway. Collectively, the results imply that EGFR activated MAP kinase signaling pathway may be involved in TLCA-induced RMCCA-1 cell growth.

## Discussion

The present study provides further understanding of the potential molecular mechanism underlying the bile acid-induced bile duct cancer development and progression. We showed that among the various forms of bile acid, TLCA can induce growth of RMCCA-1 cells via EGFR/ERK1/2 signaling pathway. Importantly, the functional presence of cholinergic system in CCA plays a certain role on this growth promoting effect of TLCA.

We found that most primary bile acids, secondary bile acids and glycine-conjugated bile acids at high concentration (100 μM) significantly decreased the viability of CCA cells. This observation is in line with a previous report showing that 100–200 μM of CA, DCA or CDCA inhibited growth of QBC939 cell, which is a human CCA cell line, by promoting cell apoptosis (23). Dai and colleagues reported that glycine-conjugated bile acids including GCA, GDCA, and GCDCA at very high concentrations (400–800 μM) stimulated growth of QBC939 cells (23). In addition, Werneburg and colleagues reported that 200 μM of DCA induced growth of KMBC which is a human CCA cell line (24). However, we did not observe the growth promoting effects of these bile acids in our tested CCA cells; this may be due to the concentration range in our study (0.1–100 μM) being far lower than the concentration range in the above mentioned studies or may be due to the difference in CCA cell lines used. Among eleven forms of bile acids, we only observed the growth promoting effect in TLCA-treated RMCCA-1 cells (Table I). Futhermore, the increased number of the S-phase cells which reflected active cell division together with the increase in level of cyclin D1, which is a key protein regulating G1/S transition in cell cycle confirmed the growth promoting effect of TLCA.

Accumulation of bile acids triggers inflammation and tumor progression (25, 26). In animal models, bile acid concentrations were increased 27-fold in liver and 1,400-fold in serum, after bile duct ligation and remained up to 14 days (27). Bile acid levels are altered in many diseases. For example, a pregnant patient who had an intrahepatic cholestasis was found to have a predominant increase in cholic acid conjugated with taurine and glycine (28). Moreover, it has been reported that the levels of glycine conjugated bile acids are increased in CCA patients (29). The exact concentration of TLCA in human liver has not been reported, but the highest level of TLCA can be found in the gallbladder and near the ampulla of Vater. Moreover, the concentration of TLCA in the gallbladder is 0.4 mM, and most TLCA is excreted in feces: a small amount of TLCA is absorbed back to enterohepatic circulation (3). There is a study that reported TLCA concentration of 2.07 pmol/mg dry weight of rat liver tissue (30). In normal situations, the ratio of glycine and taurine conjugates is at ~3:1, but in cholestasis taurine conjugation is increased (2). Moreover, TLCA has been reported to increase in the serum of cirrhotic patients (31). Therefore, it is possible that the concentration range of TLCA used in this study may be found in CCA patients.

It has been documented that M3 mAChR plays an important role in the differentiation and metastasis of CCA (18). By using a non-selective mAChR antagonist, atropine, we found that the activation of mAChR plays a crucial role in the growth promoting effect of TLCA in RMCCA-1 cells. In line with a previous colon cancer H508 cell study which overexpressed M3 mAChR, TLCA was found to interact with M3 mAChR, thereby causing an increase in inositol triphosphate 3 (IP_3_) and cell proliferation (8). Furthermore, it has been reported that TLCA can bind with M3 AChR but cannot bind to other types of mAChR in Chinese hamster ovary (CHO) cells (9). The differential sensitivity of the CCA cell lines to TLCA-induced cell growth could be explained in part due to the difference in molecular characteristics of the different CCA cell lines. The growth promoting effect of TLCA was not evidenced in HuCCA-1 cells. We found that the cholinergic components, including CHT, ChAT, AChE, M3 mAChR and α7 nAChR, were present in both HuCCA-1 and RMCCA-1 cells. The cholinergic responses to mAChR agonists, including carbachol and Oxo-M were only evidenced in RMCCA-1 cells. It is reasonable to postulate that the presence of functional cholinergic system in CCA cells may explain the different growth promoting response of TLCA. On the other hand, a previous study in QBC939 cells showed that pilocarpine, a non-selective mAChR agonist, inhibits cell proliferation while atropine can reverse this inhibitory effect (18). This opposite result may depend on cell types, mutation patterns of mAChR, and experimental design. It should be emphasized that signaling pathway involving receptors are in a dynamic state. Therefore, time course of exposure and the concentration used are important.

It is well documented that cholinergic system plays an important role in inflammation; the blockage of mAChR produced anti-inflammation properties in LPS-induced lung inflammation (32). Furthermore, selective mAChR antagonists have been used to treat many diseases such as skin inflammatory disorders, asthma, intestinal inflammation and systemic inflammation diseases (33). Moreover, our results showed that COX-2, a key inflammatory marker protein, in these two cell lines is different. RMCCA-1 showed a low level of COX-2 while HuCCA-1 showed a high level. Therefore, the inflammation background of CCA may influence the functional cholinergic system which involves the response of TLCA. However this hypothesis remains inconclusive and needs to be further investigated.

There are studies indicating that bile acids stimulate cell signaling and cell growth through the EGFR (4). It has been reported that DCA can induce caudal homeobox gene 2 (CDX2) through activation of EGFR in human mucosal epithelial SEG-1 cells (34). Moreover, there are reports of bile acids, including DCA, CDCA and TCDCA, induced cell growth and EGFR activation by the transforming growth factor-α (TGF-α), ligand-dependent mechanism in human CCA KMBC and normal cholangiocyte H69 cell lines (24). By using the specific EGFR inhibitor AG1478, we made it clear that EGFR/ERK1/2 signaling pathway is involved in the growth promoting effect of TLCA in RMCCA-1 cells. This finding is related to a previous report by Cheng and Raufman showing that conjugated secondary bile acids, including TLCA, TDCA and GDCA stimulate colon cancer H508 cell proliferation by activation of EGFR and post-EGFR/ERK1/2 signaling pathway (4).

It has been reported that TLCA induced growth of colon cancer cells through the M3 mAChR-transactived EGFR signaling pathway (10). Our study showed that atropine could not prevent the phosphorylation of EGFR and ERK1/2-induced by TLCA at 1 h of exposure (Fig. 7), suggesting that M3 mAChR may not transactivate EGFR in RMCCA-1 cells. However, both atropine and AG1478 completely inhibited the growth stimulating effect of TLCA (Figs. 6 and 8). Therefore, the transactivation of EGFR by mAChR cannot be ruled out. More selective M3 mAChR antagonist or time course studies on the effect of atropine (a non-selective mAChR antagonist) on the activation of EGFR and ERK1/2 are required.

**Figure 10:**
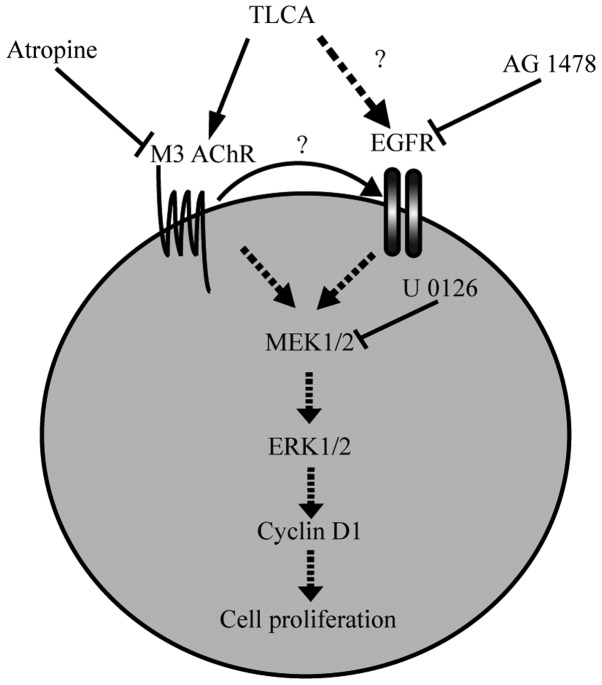
Proposed diagram of signaling pathways of TLCA mediated RMCCA-1 proliferation. TLCA binds to M3 AChR, which can be blocked by a non-selective mAChR antagonist (atropine). TLCA activates EGFR, which can be blocked by EGFR kinase inhibitor (AG 1478). Activation of M3 AChR and EGFR resulted in activated downstream MAP kinase signaling, which can be blocked by MEK 1/2 inhibitor (U 0126).

The present study provides evidence of the TLCA mechanism that activates CCA cell proliferation and which may provide a basis for therapeutic strategies to treat CCA patients. The results of the study suggest that TLCA induces the proliferation of CCA via mAChR and EGFR/ERK1/2 signaling pathway (Fig. 10). Moreover, the presence of functional cholinergic system and inflammation background of CCA plays a crucial role in the growth promoting effect of TLCA.

## Figures and Tables

**Table I:** The effects of bile acids and metabolites on cell viability.

	Cell viability (% of control)							
	
	RMCCA-1 concentration (μM)				HuCCA-1 concentration (μM)			
		
Bile acids and metabolites	0.1	1	10	100	0.1	1	10	100
Cholic acid (CA)	104.2±4.2	105.0±3.8	104.7±2.6	100.5±2.2	103.0±2.5	100.6±2.6	100.6±1.4	88.4±3.6^a^
Chenodeoxycholic acid (CDCA)	104.6±0.7	100.4±1.1	96.8±2.8	51.8±3.8^a^	103.1±2.4	104.8±1.9	100.2±3.2	49.9±2.8^a^
Deoxycholic acid (DCA)	106.8±1.6	107.3±1.1	102.3±8.5	48.4±2.1^a^	93.5±6.6	95.0±0.7	86.2±6.1	27.4±1.0^a^
Lithocholic acid (LCA)	104.6±0.7	104.0±2.9	56.3±1.0^a^	7.5±0.8^a^	106.7±1.0	103.9±4.0	55.2±5.9^a^	5.0±0.7^a^
Glycine conjugated bile acids								
Glycocholic acid (GCA)	94.6±2.7	93.9±0.7	93.1±1.7	85.3±2.9^a^	106.1±2.1	94.7±1.1	93.3±0.2	82.0±3.6^a^
Glycochenodeoxycholic acid (GCDCA)	102.0±1.0	98.2±1.6	94.3±6.5	83.5±7.3	106.5±2.1	100.6±1.7	92.3±1.2^a^	77.7±2.7^a^
Glycodeoxycholic acid (GDCA)	97.5±2.5	91.6±2.1	94.7±2.3	86.7±1.4^a^	105.2±4.1	104.2±5.1	98.4±2.4	77.5±0.1^a^
Taurine conjugated bile acids								
Taurocholic acid (TCA)	100.4±3.2	98.4±3.7	99.7±5.6	95.5±5.4	108.1±3.2	106.9±2.9	107.3±4.0	94.4±3.2
Taurochonodeoxycholic acid (TCGCA)	95.3±3.6	99.7±3.8	95.5±4.9	84.6±8.0	98.7±2.0	96.8±3.3	91.1±3.2	73.4±6.6^a^
Taurodeoxycholic acid (TDCA)	100.8±1.2	102.6±1.7	100.4±3.3	95.2±2.7	106.5±2.5	105.7±2.2	102.2±2.9	89.5±5.2
Taurolithocholic acid (TLCA)	109.8±1.7	112.0±8.2	118.3±1.3^a^	80.1±6.2^a^	95.1±1.6	98.1±1.8	94.4±2.5	55.3±7.1^a^

Values are mean ± SE.

a p<0.05.
